# Influence of early-life parental severe life events on the risk of type 1 diabetes in children: the DiPiS study

**DOI:** 10.1007/s00592-018-1150-y

**Published:** 2018-05-12

**Authors:** Markus Lundgren, Katarina Ellström, Helena Elding Larsson, for the DiPiS study group, C. Andersson, R. Bennet, I. Jönsson, M. Ask, J. Bremer, C. Brundin, C. Cilio, C. Hansson, G. Hansson, S. Ivarsson, B. Jonsdottir, Å Lernmark, B. Lindberg, B. Lernmark, Å. Lernmark, Z. Mestan, A. Ramelius, U.-M. Carlsson, A. Carlsson, E. Cedervall, B. Jönsson, K. Larsson, J. Neiderud

**Affiliations:** 1 Unit for Pediatric Endocrinology, Department of Clinical Sciences Malmö, Lund University, Malmö, Sweden; 2 Department of Pediatrics, Kristianstad Central Hospital, Kristianstad, Sweden; 3 Pediatric Endocrinology and Gastroentetology, Skåne University Hospital, Malmö, Sweden

**Keywords:** Diabetes mellitus, Type 1, Prospective studies, Stress, Psychological, Pediatrics

## Abstract

**Aims:**

Stress and severe life events (SLEs) modify autoimmune disease susceptibility. Here, we aimed to establish if SLEs reported by parents during the first 2 years of life influence the risk of developing type 1 diabetes (T1D) using data from the prospective Diabetes Prediction in Skåne (DiPiS) study.

**Methods:**

Prospective questionnaire data recorded at 2 months (*n* = 23,187) and 2 years of age (*n* = 3784) from the DiPiS cohort of children were included in the analysis. SLEs were analyzed both by groups and as a combined variable. A Cox proportional hazards model was used to calculate hazard ratios (HRs) for T1D diagnosis for the total cohort and for the HLA-DQ2/8 high-risk population. Affected first-degree relatives, HLA-DQ risk group, paternal education level, and parents’ country of birth were included as covariates.

**Results:**

There was a significantly increased risk of T1D in children with SLEs occurring during the child’s first 2 years of life for both the total cohort (HR 1.67; 95% CI 1.1, 2.7; *p* = 0.03) and the DQ2/8 cohort (HR 2.2; 95% CI 1.1, 4.2; *p* = 0.018). Subgroup analysis of events related to unemployment, divorce, or family conflict showed a significant hazard for these events occurring both during and after pregnancy in the DQ2/8 cohort (HR 2.17; 95% CI 1.1, 4.3; *p* = 0.03 and HR 4.98; 95% CI 2.3, 11; *p* < 0.001, respectively) and after pregnancy in the total cohort (multiple regression HR 2.07; 95% CI 1.01, 4.2; *p* = 0.047).

**Conclusions:**

Children of parents experiencing an SLE during the child’s first 2 years of life were at increased risk of T1D. Further studies including those measuring immune and stress-related biomarkers are necessary to validate the findings.

## Introduction

The potential influence of psychological stress on type 1 diabetes (T1D) is well studied. Stress is associated with systemic immunological and hormonal changes that can alter the immunological balance to increase the risk of autoimmunity and T1D via a number of different mechanisms [1–4].

Elevated cortisol levels represent an acute response to stress that has immediate effects on both the immune system and metabolism [5–7]. Additionally, there are data supporting long-term effects of early elevated cortisol levels on the risk of immunological diseases and allergies [8, 9]. Cortisol also appears to have a direct effect on insulin resistance, adding to the strain on pancreatic beta cells [10]. Children exposed to early life stress have been shown to have higher C-reactive protein levels than average as adults, and adults with a history of childhood abuse have higher levels of circulating pro-inflammatory cytokines than the general population [9, 11].

One distinct psychological stressor is a severe life event (SLE) such as a bereavement, serious family illness, family conflict, and socio-economic problems [12]. Early SLEs have been linked to an increased risk of T1D and islet autoimmunity [10, 13–15], although data are conflicting [13–15], with very young individuals apparently more vulnerable to the immunomodulatory effects of SLEs than older children and adults [16]. A growing body of evidence proposes that the ability of the individual to cope with stress and adversity may also be affected by factors that can be estimated using quality of life or sense of coherence measures [17, 18]. A high quality of life/sense of coherence may, therefore, act as a buffer, decreasing the negative impact of the event(s).

The stress levels of infants and children are hard to quantify but have been found to be correlated with the stress levels of their parents. Furthermore, like many other risk factor studies, there is a long latency between exposure and T1D disease onset. Nevertheless, there are new data on the early stages of T1D development that indicate that very early factors associated with autoimmune processes leading to islet autoimmunity and eventually T1D are relevant [19, 20].

The Diabetes Prediction in Skåne (DiPiS) study is a prospective follow-up of children at risk of T1D in southern Sweden. Here, we investigated the effects of early-life family SLEs on the risk of T1D using questionnaire data recorded by parents when the child was between 2 months and 2 years of age. We also evaluated if children with the highest HLA-conferred risk (HLA DQ2/8) were more susceptible to stress compared to children with a lower genetic T1D risk.

## Methods

### The DiPiS study

Between August 2000 and September 2004, 48,058 children were born in the five hospitals in the Skåne region in southern Sweden. 35,683 cord blood samples were collected at birth along with basic demographic data. Cord blood samples were analyzed for autoantibodies to glutamate decarboxylase 65 (GAD65A), insulin (IAA), insulinoma antigen 2 (IA-2A), and HLA-DQ genotype [21, 22]. At 2 months of age, parents of study participants were asked to complete a questionnaire regarding perinatal, hereditary, medical, and social factors, as well as if the parent had experienced an SLE during pregnancy or the child’s first 2 months of life. 23,187 questionnaires with written consent for study participation were returned.

A basic risk model was developed accounting for HLA-conferred T1D risk, infections during pregnancy, deviating relative birth weight, affected first-degree relatives, and cord blood autoantibody positivity. Based on this risk model, 7826 participants were invited for follow-up, of whom 3784 accepted and returned a questionnaire at 2 years of age including information regarding medical history, psychosocial setting, parental SLEs, and questions regarding study participation during the child’s first 2 years of life.

From the age of two, study participants were screened for islet autoantibodies and were administered questionnaires on a yearly basis. These questionnaires include a wide range of questions regarding the lives of the child and family including dietary factors, hospitalization, illness and medications, psychosocial factors, child care, disease in the immediate family, and whether any parent has experienced a severe life event. Participants who developed two or more positive autoantibodies were offered follow-up every 3 months with autoantibody analysis for GAD65A, IAA, IA-2A and three zinc transporter 8 (ZnT8A) isoforms, HbA1c, random plasma glucose, growth parameters, and annual oral glucose tolerance test. Data regarding T1D heredity were collected from both a form from the maternity department at birth and the 2-month and 2-year questionnaires, the questions asked being if the parent or siblings had diabetes and if they were treated with insulin. No follow-up question regarding the type of diabetes was asked at this time.

**Fig. 1:**
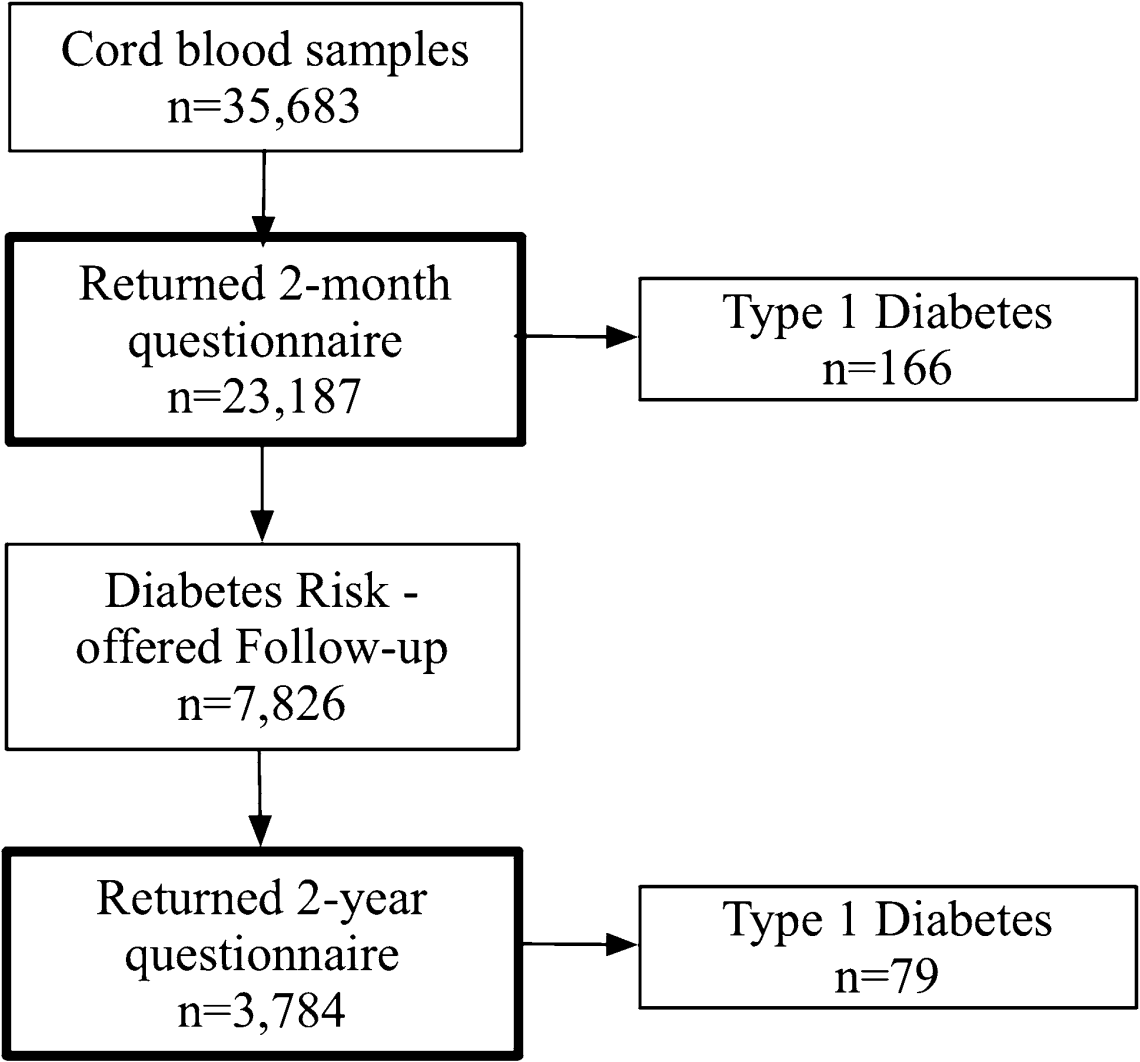
Study inclusion flow chart

Insulin-dependent diabetes mellitus (IDDM) in a first-degree relative was defined as the mother, father, or any sibling being treated with insulin for diabetes. Mothers reported to have gestational diabetes or diabetes but not treated with insulin were excluded from the IDDM group. Incidence data regarding T1D were collected through the participating clinics and via the Better Diabetes Diagnosis (BDD) study, which covers approximately 95% of children diagnosed with T1D in Sweden [23]. All children with answers to the 2-month and 2-year questionnaires were included in the study (Fig. 1).

The study was approved by the Regional Ethics review board in Lund.

### Severe life event analysis

The 2-month questionnaire contained questions regarding life events during pregnancy and during the child’s first 2 months of life. The question “Did you experience anything that you would consider to be a significant life event during pregnancy or after the child’s birth?” was followed by eight options: “death of a close relative”; “severe disease in the family”; “serious accident in the family”; “own divorce or separation”; “been subjected to violence”; “lost employment”; “husband/partner lost employment”; “serious conflict with partner or other significant person”. The same questions were repeated in the 2-year questionnaire.

Due to the relatively small number of events in each individual category, two larger combined variables were also analyzed for SLEs: (1) events of a more acute and sudden nature, including severe disease in the family, death of a close relative, serious accident in the family, or having experienced violence; and (2) SLEs of a lower intensity and possibly longer duration including divorce in the family, either parent becoming unemployed, or divorce of the parents.

All SLEs during pregnancy were analyzed as a separate outcome. For SLEs after birth during the child’s first 2 years of life, the answers from the 2-month and 2-year questionnaires were combined into a joint outcome variable. Thus, this variable incorporates SLEs during the child’s first 2 months and/or 2 years of life. Analysis was also performed on a combined variable constructed to indicate if any of the respective questions had been indicated as “yes” either during or after pregnancy and combined as “life event during pregnancy” and “life event after pregnancy”.

### Statistical analysis

Potential covariates were selected a priori from the available data derived from the questionnaires. These were factors with theoretical influence on the impact of SLEs (parental education level, parent born outside of Sweden, and maternal age) or on T1D risk (HLA risk group, first-degree relative diabetes status, and gender). A univariable Cox proportional hazards model was used to assess possible covariates for the multiple regression analysis. Adherence to the proportional hazards assumption for all covariates was analyzed using scaled Schoenfeld residuals. Models fitted including the a priori-selected possible covariates, taking account of the Wald statistic, likelihood ratio, and Akaike information criterion (AIC) for the respective models. The total cohort was then evaluated using a Cox multiple regression model with HLA risk group [DQ2/8, DQ8/8 or DQ8/X, DQ2/2 or DQ2/X, and DQX/X (X denoting neither 8 nor 2)], first-degree relative with insulin-dependent diabetes (yes/no), mother born in Sweden (yes/no), and father born in Sweden (yes/no) included as model covariates.

A separate analysis was performed for the high-risk DQ2/8 sub-cohort. Similar to the full cohort, model fit was evaluated using the Wald statistic, likelihood ratio, and model AIC. In this analysis, the final Cox proportional hazards model included only first-degree relative with insulin-dependent diabetes (yes/no) as a covariate. To maximize the use of questionnaire data, all available cases were included in the analysis, and missing data were excluded by pairwise deletion. All cases that had not developed T1D were censored with the date set at November 30, 2017. Statistical analysis was performed using R version 3.4.4 with the Survival (version 2.41) and Survminer (version 0.4.2) packages (R Foundation for Statistical Computing, Vienna, Austria; https://www.R-project.org).

## Results

### Baseline characteristics

The parents of 23,187 children answered the 2-month questionnaire and 3784 answered the 2-year questionnaire. The mean follow-up for study participants was 15.1 years (SD 1.27 years), and 166 children were diagnosed with T1D in the study cohort. The mean age at T1D diagnosis was 8.54 years (SD 4.03 years) for the 166 children with a T1D diagnosis in the total cohort and 7.71 years (SD 4.08) for the 61 children with T1D in the HLA-DQ2/8 sub-cohort. In the 2-month questionnaire, 4,361 (18.9%) parents reported having experienced an SLE during pregnancy, and 2,311 (10.0%) experienced an SLE during the child’s first 2 months of life. In the 2-year questionnaire, 1234 (32.6%) parents reported having experienced SLEs since the last questionnaire was returned. The characteristics of 2-month respondents and 2-year respondents are shown in Table 1. Significant factors entered into a Cox multiple regression analysis included mother born in Sweden (HR 2.4; 95% CI 1.2, 4.8; *p* = 0.016), father born in Sweden (HR 2.97; 95% CI 1.3, 6.7; *p* = 0.009), and first-degree relative with IDDM (HR 6.98; 95% CI 4.3, 11; *p* < 0.001). All univariate results on both the total and DQ2/8 cohorts are presented in Table 2.

**Table 1:** Characteristics of respondents to the 2-month and 2-year questionnaire

		2-month questionnaire (*n* = 23,187)	2-year questionnaire (*n* = 3861)
Maternal age at birth	Mean (IQR)	30.8 (6.0)	31.4 (6.0)
Male child	*n* (%)	11,589 (51.6)	1899 (52.2)
Gestational age	Median (IQR)	39.3 (2.0)	39.1 (2.0)
First-degree relative IDDM	*n* (%)	430 (1.9)	259 (6.9)
HLA			
DQ2/8	*n* (%)	865 (3.7)	431 (11.4)
DQ8/8 or 8/X	*n* (%)	2163 (9.3)	1140 (30.1)
DQ2/2 or 2/X	*n* (%)	1937 (8.4)	1016 (26.8)
DQ X/X	*n* (%)	18,190 (78.6)	1197 (31.6)
Maternal education			
9-year compulsory school	*n* (%)	1412 (6.1)	196 (5.2)
Senior high school	*n* (%)	11,611 (50.4)	1812 (48.1)
College/university	*n* (%)	10,028 (43.5)	1761 (46.5)
Paternal education			
9-year compulsory school	*n* (%)	2079 (10.0)	336 (9.6)
Senior high school	*n* (%)	11,411 (49.2)	1885 (53.7)
College/university	*n* (%)	7379 (35.4)	1287 (36.7)
Mother born in Sweden	*n* (%)	20,419 (89.2)	3472 (93.0)
Father born in Sweden	*n* (%)	18,553 (88.8)	3434 (92.1)
Severe life events after birth	*n* (%)	2311 (10.0)	1234 (32.6)

**Table 2:** Hazard ratios for baseline demographic factors in the total and HLA-DQ2/8 cohort

	Total cohort (*n* = 23,187)			HLA-DQ2/8 cohort (*n* = 865)		
	HR	95% CI	*p*	HR	95% CI	*p*
Maternal age	1.00	0.97:1.0	0.88	1.02	0.97:1.1	0.48
Sex	0.77	0.56:1.1	0.10	0.80	0.48:1.3	0.39
Gestational length^a^	0.81	0.59:1.1	0.21	0.95	0.83:1.1	0.51
Relative birth-weight quartile	1.00	0.99:1.0	0.97	0.99	0.99:1.0	0.49
Maternal education level^b^	0.84	0.65:1.07	0.16	0.89	0.59:1.4	0.59
Paternal education level^b^	0.76	0.59:0.98	0.032	0.70	0.46:1.1	0.09
Mother born in Sweden	2.40	1.2:4.8	0.016	2.83	0.69:11	0.15
Father born in Sweden	2.97	1.3:6.7	0.009	1.21	0.44:3.4	0.71
First-degree relative IDDM^c^	6.98	4.3:11	< 0.001	2.54	1.15:5.6	0.02
HLA haplotype						
DQ2/8	42.4	27:66	< 0.001			
DQ8/8 or DQ8/X	13.1	8.2:21	< 0.001			
DQ2/2 or DQ2/X	7.19	4.1:13	< 0.001			
DQ X/X	Ref	Ref	Ref			

*Ref* reference level chosen in the analysis

^a^Gestational week group (< 37, 37–40, > 40 weeks)

^b^Education level: Primary school, high school, college, university

^c^First-degree relative with diabetes treated with insulin

### Severe life events

**Fig. 2:**
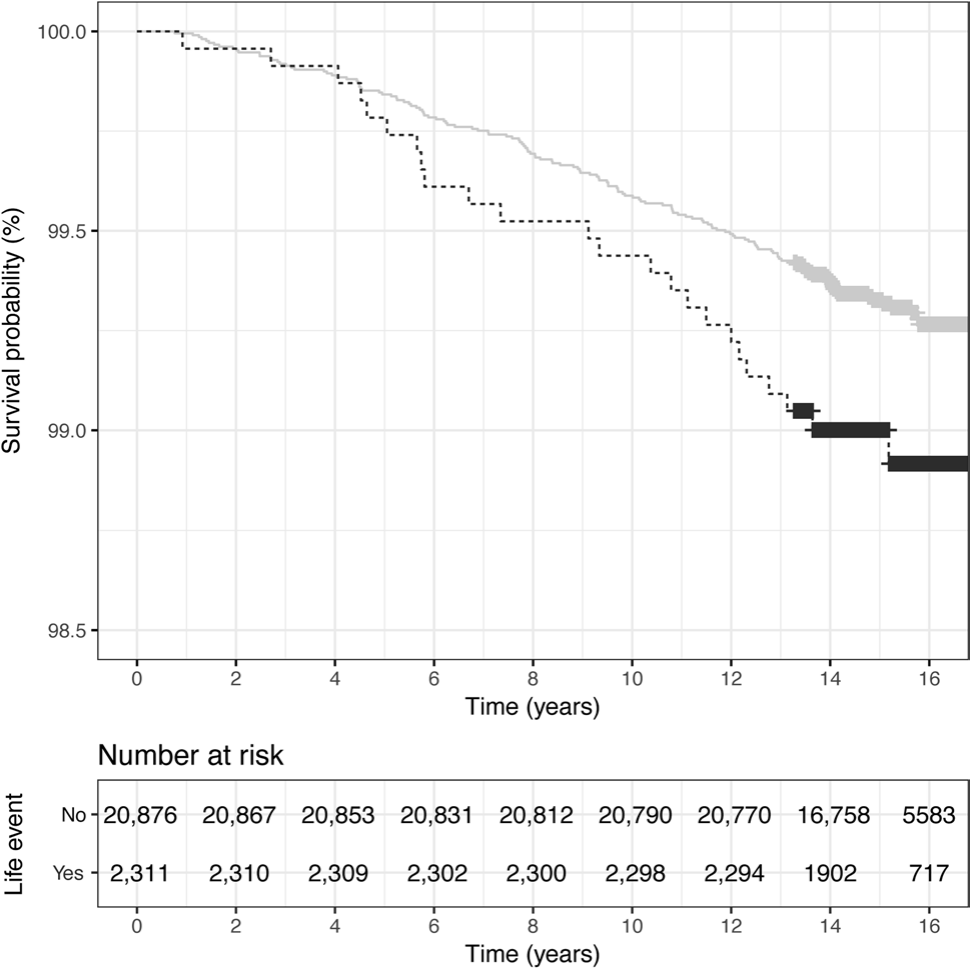
Kaplan–Meier curves of SLEs after pregnancy for the total cohort. Survival probability is plotted on the *y*-axis; note that this axis does not start at zero. Log-rank test: *χ*^2^ (1, *n* = 23,187) = 3.7, *p* = 0.055. Gray line: not exposed to event; black line: exposed to event

**Fig. 3:**
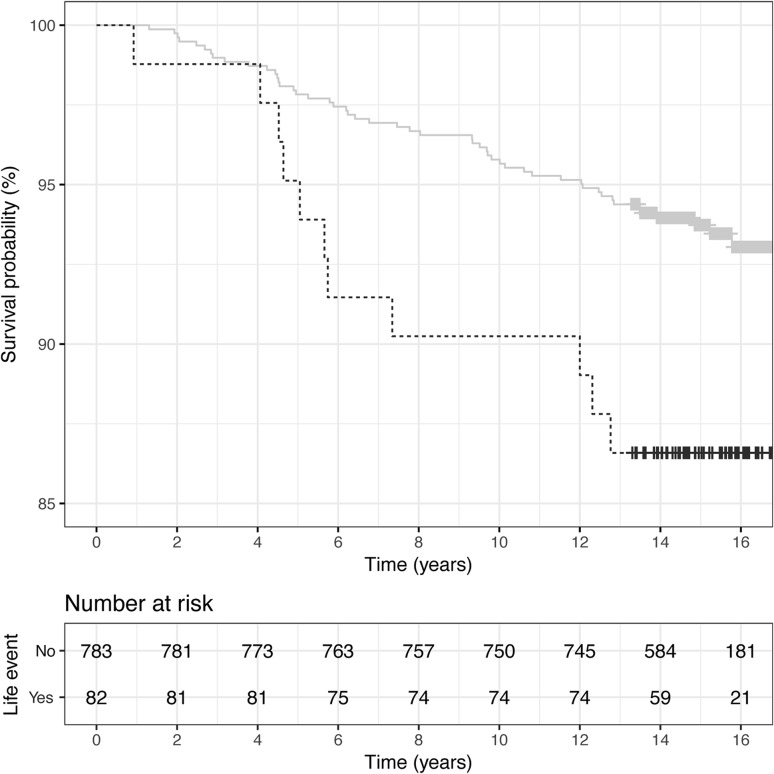
Kaplan–Meier curves of SLEs after pregnancy for the DQ2/8 cohort. Survival probability is plotted on the *y*-axis; note that this axis does not start at zero. Log-rank test: *χ*^2^ (1, n = 865) = 5.8, *p* = 0.016. Gray line: not exposed to life event; black line: exposed to event

The number and proportion of SLEs for children diagnosed with T1D are shown in Table 3. In the total cohort, there was a significantly increased risk of T1D if the parents reported an SLE between birth and 2 years of age (multiple regression HR 1.67; 95% CI 1.1, 2.7; *p* = 0.03). Kaplan–Meier curves of SLEs after pregnancy for the total cohort including number of subjects at risk is shown in Fig. 2 and for the DQ2/8 cohort in Fig. 3. There was also an increased risk for children whose parents reported unemployment/conflict in the family or divorce during the child’s first 2 years of life (multiple regression HR 2.07; 95% CI 1.01, 4.2; *p* = 0.047). There was no increased risk of T1D in children whose parents had experienced an SLE during pregnancy (multiple regression HR 1.27; 95% CI 0.85, 1.9; *p* = 0.24).

**Table 3:** Hazard ratios for SLE in the total cohort and the HLA-DQ2/8 study cohort

	Exposed to event		Univariable Cox regression			Multivariable Cox regression		
	Diabetes cases [*n* (%)]	Controls [*n* (%)]	HR	95% CI	*p*	HR	95% CI	*p*
Total cohort^a^ (*n* = 23,187)								
SLE, during pregnancy	38 (22.9)	4323 (18.8)	1.28	0.89:1.8	0.20	1.27	0.85:1.9	0.24
SLE, after pregnancy	24 (14.5)	2287 (9.9)	1.52	0.99:2.3	0.06	1.67	1.1:2.7	0.03
SLE, during and/or after pregnancy	51 (30.7)	5521 (24.0)	1.40	1.00:1.9	0.045	1.39	0.97:2.0	0.07
Disease/death/accident/violence, during pregnancy	21 (12.6)	2451 (10.7)	1.21	0.77:1.9	0.68	1.13	0.69:1.9	0.62
Disease/death/accident/violence, after pregnancy	11 (6.6)	1211 (5.3)	1.27	0.69:2.3	0.57	1.32	0.69:2.5	0.40
Unemployment/conflict/divorce, during pregnancy	18 (10.8)	1844 (8.0)	1.40	0.86:2.3	0.18	1.59	0.92:2.8	0.10
Unemployment/conflict/divorce, after pregnancy	10 (6.0)	898 (3.9)	1.58	0.83:3.0	0.16	2.07	1.01: 4.2	0.047
HLA-DQ 2/8 cohort^b^ (*n* = 865)								
Serious life event, during pregnancy	16 (17.2)	156 (18.7)	1.45	0.82:2.6	0.20	1.37	0.76:2.5	0.29
Serious life event, after pregnancy	11 (11.8)	71 (8.5)	2.19	1.1:4.2	0.019	2.2	1.1:4.2	0.018
Serious life event, during and/or after pregnancy	20 (21.5)	197 (23.6)	1.48	0.87:2.5	0.15	1.41	0.82:2.4	0.21
Disease/death/accident/violence, during pregnancy	8 (8.6)	89 (10.6)	1.19	0.57:2.5	0.64	1.05	0.47:2.3	0.90
Disease/death/accident/violence, after pregnancy	3 (3.2)	39 (4.7)	1.01	0.32:3.2	0.63	1.00	0.31:3.2	0.99
Unemployment/conflict/divorce, during pregnancy	10 (10.8)	69 (8.2)	2.02	1.0:4.0	0.042	2.16	1.1:4.3	0.03
Unemployment/conflict/divorce, after pregnancy	7 (7.5)	19 (2.3)	4.71	2.1:10	< 0.001	4.98	2.3:11	< 0.001

^a^Cox multiple regression model: first-degree relative IDDM, mother born in Sweden, father born in Sweden and HLA risk group included as model covariates

^b^Cox multiple regression model: first-degree relative IDDM included as model covariate

In the DQ2/8 cohort, children with parents reporting an SLE during the child’s first 2 years of life had a significantly increased hazard ratio for T1D (HR 2.2; 95% CI 1.1, 4.2; *p* = 0.018), as did those whose parents reported unemployment/conflict in the family or divorce during pregnancy (HR 2.16; 95% CI 1.1, 4.3; *p* = 0.03) or during the child’s first 2 years of life (HR 4.98; 2.3, 11; *p* < 0.001).

## Discussion

In this study, we investigated the effects of self-reported, parental SLEs occurring during pregnancy and early life on T1D risk. There was an increased T1D risk for children in families reporting an SLE during the child’s first 2 years of life, both in the population as a whole (HR 1.7) and in individuals with the high-risk HLA DQ2/8 haplotype (HR 2.2). Children whose parents reported unemployment, family conflict, or divorce after pregnancy in both cohorts (total cohort HR 2.1; DQ2/8 cohort HR 4.98) but also during pregnancy in the DQ2/8 cohort were at significantly increased risk of T1D (HR 2.16).

Several studies have examined the relationships between living conditions, stress levels, and immune system function. Chronic or long-term stress is correlated with increased CRP levels [11], altered cortisol levels [6, 7, 24], and a dysregulated, pro-inflammatory, immune response [4, 8, 25, 26]. There is also a correlation between stress levels in parents and levels of cortisol secretion in their young children [5, 27, 28].

The potential for stress and family SLEs to contribute to autoimmunity or progression to clinical T1D has long been suspected. Stress was proposed to be a major contributor or the main culprit of T1D pathogenesis in early studies [3], but since then our understanding of the etiology of T1D and islet autoimmunity has increased greatly to reveal a complex, multifactorial pathoetiology [29–31]. Despite this, however, stress and SLE remain potential prenatal and postnatal T1D risk factors.

Prenatal stress has been shown to increase T1D risk [32], but these results are disputed [14]. The impact of stress and SLEs on T1D risk has been more thoroughly studied [13, 15, 33]. Our finding that SLEs occurring during a child’s first years of life increase the risk of T1D is consistent with previous studies [2, 34]. The interpretation of earlier studies on the effects of stress on T1D risk is in many cases hard considering our current understanding of T1D pathoetiology. Many of these earlier studies focused on the near pre-diagnostic period, at which point we can assume that islet autoimmunity and possibly dysglycemia are already established. Hence, the increased risk reported in these studies most likely represents an increase in the progression rate from presymptomatic to symptomatic T1D and not an increase in T1D risk per se [35–37].

In this study, we analyzed both the total available cohort and a sub-cohort comprised only of children with the HLA DQ2/8 high-risk genotype. The rationale for this analysis was threefold. First, we hypothesized that the children at the highest genetic risk of T1D may also be most susceptible to the effects of SLEs. Second, we aimed to create a sub-cohort of children with a homogenous genetic risk profile to minimize the effects of HLA on the results. Third, previously published data did not include HLA risk in the statistical analysis, so this analysis was novel [38]. Our results indicate that in the HLA DQ2/8 sub-cohort the risk conferred by experiencing an SLE is higher (HR 2.31; *p* = 0.016) than in the total cohort (HR 1.65; *p* = 0.035), with a higher level of significance despite a much smaller number of children in the analysis (total *n* = 23,187; HLA DQ2/8 *n* = 865).

That both the 2-month and 2-year questionnaires were used introduces some bias into the analysis. The questionnaires covered dissimilar timeframes, with 10% of the respondents to the 2-month questionnaire and 33% of the respondents to the 2-year questionnaire reporting SLEs. Additionally, the questionnaires were answered by 23,187 and 3861 families, respectively. The method of data collection must be taken into account in the interpretation of our results.

This study is limited by its reliance on prospectively collected questionnaire data. The DiPiS study protocol did not include a validated questionnaire on parental or child stress, and during the early part of the study no questions were asked regarding whether the child was affected by SLEs and to what degree the child was affected. The use of a validated stress scale would have greatly strengthened the interpretation of the results of this study. Evaluation of psychosocial factors that may affect the impact of negative life events would have improved the weight of the study’s results. A measure of quality of life or sense of coherence, both factors that affect the impact of negative life events, would, in hindsight, have been valuable tools to further understand the present results [17, 18].

The number of SLEs in the cohort was low, despite the fairly large number of participants, weakening the statistical analysis. There were no laboratory data such as cortisol levels or other immune biomarkers to support the results. The 2-month questionnaire was returned by 65% of parents of the originally screened children and the 2-year questionnaire by 49% of parents of the high-risk children invited to participate in the study. However, the latter respondents only amounted to 11% of children initially screened. Regarding the generalizability of the results, the respondents to the 2-month questionnaire were estimated to be similar to the general population but with the usual risk of having an excess of answers from well-functioning families. The same risk applied to the 2-year questionnaire, which was further complicated by this cohort being an at-risk cohort only. These factors need to be accounted for in the interpretation of the results.

Nevertheless, the DiPiS study is a large-scale, prospective follow-up study incorporating a large proportion of at-risk children born between 2000 and 2004 in southern Sweden and is now almost complete with 14 years mean follow-up. This, combined with excellent prevalence data regarding T1D derived from the BDD study, is a major strength of this study. We also analyzed answers from both parents, not only the mother, in contrast to several earlier studies, improving the overall data quality and minimizing data loss due to recall problems.

In conclusion, there was an increased risk of T1D in children whose parents reported being exposed to SLEs during the two first years of life. These data add to our understanding of the potential contribution of stressful events and psychosocial factors to T1D pathoetiology. However, due to the small number of events, the results need to be interpreted with care. To fully understand the effects of stress on autoimmunity and T1D risk, additional well-planned, large-scale studies with validated study questionnaires and laboratory data will be of importance.

## Abbreviations

DiPiS: Diabetes Prediction in Skåne
SLE: Severe life event
T1D: Type 1 diabetes
BDD: Better Diabetes Diagnosis
IDDM: Insulin-dependent diabetes mellitus

## References

[1] Nicolaides NC, Kyratzi E, Lamprokostopoulou A (2014). Stress, the stress system and the role of glucocorticoids. Neuroimmunomodulation.

[2] Thernlund GM, Dahlquist G, Hansson K (1995). Psychological stress and the onset of IDDM in children. Diabetes Care.

[3] Stein SP, Charles E (1971). Emotional factors in juvenile diabetes mellitus: a study of early life experience of adolescent diabetics. Am J Psychiatry.

[4] Harpaz I, Abutbul S, Nemirovsky A (2013). Chronic exposure to stress predisposes to higher autoimmune susceptibility in C57BL/6 mice: glucocorticoids as a double-edged sword. Eur J Immunol.

[5] Koch F-S, Ludvigsson J, Sepa A (2010). Parents’ psychological stress over time may affect children’s cortisol at age 8. J Pediatr Psychol.

[6] Gustafsson PE, Gustafsson PA, Nelson N (2006). Cortisol levels and psychosocial factors in preadolescent children. Stress Health.

[7] Carpenter LL, Carvalho JP, Tyrka AR (2007). Decreased adrenocorticotropic hormone and cortisol responses to stress in healthy adults reporting significant childhood maltreatment. Biol Psychiatry.

[8] Miller GE, Cohen S, Ritchey AK (2002). Chronic psychological stress and the regulation of pro-inflammatory cytokines: a glucocorticoid-resistance model. Health Psychol.

[9] Faresjo M (2015). The link between psychological stress and autoimmune response in children. Crit Rev Immunol.

[10] Sepa A, Wahlberg J, Vaarala O (2005). Psychological stress may induce diabetes-related autoimmunity in infancy. Diabetes Care.

[11] Broyles ST, Staiano AE, Drazba KT (2012). Elevated C-reactive protein in children from risky neighborhoods: evidence for a stress pathway linking neighborhoods and inflammation in children. PLoS One.

[12] Sepa A, Ludvigsson J (2007). Psychological stress and the risk of diabetes-related autoimmunity: a review article. Neuroimmunomodulation.

[13] Cosgrove M (2004). Do stressful life events cause type 1 diabetes?. Occup Med (Lond).

[14] van Dijk AE, van Eijsden M, Stronks K (2014). No associations of prenatal maternal psychosocial stress with fasting glucose metabolism in offspring at 5–6 years of age. J Dev Orig Health Dis.

[15] Littorin B, Sundkvist G, Nyström L (2001). Family characteristics and life events before the onset of autoimmune type 1 diabetes in young adults: a nationwide study. Diabetes Care.

[16] Coe CL, Lubach GR (2003). Critical periods of special health relevance for psychoneuroimmunology. Brain Behav Immun.

[17] Eriksson M, Lindström B (2006). Antonovsky’s sense of coherence scale and the relation with health: a systematic review. J Epidemiol Community Health.

[18] The WHO QoL Working Group (1995). The World Health Organization Quality of Life assessment (WHOQOL): position paper from the World Health Organization. Soc Sci Med.

[19] Krischer JP, Lynch KF, Schatz DA (2015). The 6 year incidence of diabetes-associated autoantibodies in genetically at-risk children: the TEDDY study. Diabetologia.

[20] Insel RA, Dunne JL, Atkinson MA (2015). Staging presymptomatic type 1 diabetes: a scientific statement of JDRF, the Endocrine Society, and the American Diabetes Association. Diabetes Care.

[21] Lynch KF, Lernmark B, Merlo J (2008). Cord blood islet autoantibodies and seasonal association with the type 1 diabetes high-risk genotype. J Perinatol.

[22] Larsson HE, Lynch K, Lernmark B (2005). Diabetes-associated HLA genotypes affect birthweight in the general population. Diabetologia.

[23] Delli AJ, Vaziri-Sani F, Lindblad B (2012). Zinc transporter 8 autoantibodies and their association with SLC30A8 and HLA-DQ genes differ between immigrant and Swedish patients with newly diagnosed type 1 diabetes in the Better Diabetes Diagnosis study. Diabetes.

[24] Ullmann E, Barthel A, Petrowski K (2016). Pilot study of adrenal steroid hormones in hair as an indicator of chronic mental and physical stress. Sci Rep.

[25] Herberth G, Weber A, Röder S (2008). Relation between stressful life events, neuropeptides and cytokines: results from the LISA birth cohort study. Pediatr Allergy Immunol.

[26] Dhabhar FS (2009). Enhancing versus suppressive effects of stress on immune function: implications for immunoprotection and immunopathology. Neuroimmunomodulation.

[27] Martin CG, Kim HK, Fisher PA (2016). Differential sensitization of parenting on early adolescent cortisol: moderation by profiles of maternal stress. Psychoneuroendocrinology.

[28] Zalewski M, Lengua LJ, Kiff CJ, Fisher PA (2012). Understanding the relation of low income to HPA-axis functioning in preschool children: cumulative family risk and parenting as pathways to disruptions in cortisol. Child Psychiatry Hum Dev.

[29] Rewers M, Ludvigsson J (2016). Environmental risk factors for type 1 diabetes. Lancet.

[30] Pociot F, Lernmark Å (2016). Genetic risk factors for type 1 diabetes. Lancet.

[31] Krischer JP, Lynch KF, Lernmark Å (2017). Genetic and environmental interactions modify the risk of diabetes-related autoimmunity by 6 years of age: the TEDDY study. Diabetes Care.

[32] Virk J, Li J, Vestergaard M (2010). Early life disease programming during the preconception and prenatal period: making the link between stressful life events and type-1 diabetes. PLoS One.

[33] Nygren M, Ludvigsson J, Carstensen J, Sepa Frostell A (2013). Family psychological stress early in life and development of type 1 diabetes: the ABIS prospective study. Diabetes Res Clin Pract.

[34] Sipetic S, Vlajinac H, Marinkovi J (2007). Stressful life events and psychological dysfunctions before the onset of type 1 diabetes mellitus. J Pediatr Endocrinol Metab.

[35] Karavanaki K, Tsoka E, Liacopoulou M (2008). Psychological stress as a factor potentially contributing to the pathogenesis of type 1 diabetes mellitus. J Endocrinol Investig.

[36] Hägglöf B, Blom L, Dahlquist G (1991). The Swedish childhood diabetes study: indications of severe psychological stress as a risk factor for type 1 (insulin-dependent) diabetes mellitus in childhood. Diabetologia.

[37] Zung A, Blumenfeld O, Shehadeh N (2012). Increase in the incidence of type 1 diabetes in Israeli children following the Second Lebanon War. Pediatr Diabetes.

[38] Nygren M, Carstensen J, Koch F (2015). Experience of a serious life event increases the risk for childhood type 1 diabetes: the ABIS population-based prospective cohort study. Diabetologia.

